# Abdominal Pain; Its Causes and Clinical Significance

**Published:** 1905-06

**Authors:** 


**Abdominal Pain; its Causes and Clinical Significance.** By A. Ernest Maylard, M.B., B.S. Pp. xii., 304. London: J. & A. Churchill. 1905.

The heading throughout is "Abdominal Pain," but about a hundred pages at the end of the book are devoted to the surgical methods which the author employs in abdominal sections. This latter part need not be considered, as it presents little beyond what is usually adopted in such cases. It is suggested that by an accurate knowledge of abdominal pain the differential diagnosis of disease will be made easy for the "busy practitioner," for "when confronted with pains, the significance of which he cannot clearly construe, he should first turn to the chapters on the regional manifestations of pain, and seek among the causes there given the possible organ affected." After that the practitioner is told to turn to the chapters containing a fuller description of the symptoms of disease in the suspected organ, and this should lead to a perusal of the whole subject in larger works. It is rather difficult to conceive a more clumsy way of arriving at a diagnosis, and the list of causes of pain in each region would require much reading before the diagnosis was reasonably accurate. This method is wrong in principle, for it gives an exaggerated importance to one symptom, and with this as a basis the judgment is likely to be warped. Tenderness rather than pain would very frequently be a much more reliable guide to the organ affected, but in any case a reasonable acquaintance with the chief symptoms of abdominal disease is more worthy of advocacy than the study of pain as of first importance from the diagnostic point of view. We have spoken from the probable degree of utility as imagined by the author, whose methods seem to be erroneous, but apart from this the work is an excellent compendium of what we know of abdominal pain. There is, however, much more to be learnt from its pages by those who are thoroughly acquainted with the symptoms of abdominal disease generally than by those less informed who endeavour to utilise its pages in the elucidation of difficult cases.

